# Prediction Factors of Recurrent Ischemic Events in One Year after Minor Stroke

**DOI:** 10.1371/journal.pone.0120105

**Published:** 2015-03-16

**Authors:** Changqing Zhang, Xingquan Zhao, Chunxue Wang, Liping Liu, Yuchuan Ding, Fauzia Akbary, Yuehua Pu, Xinying Zou, Wanliang Du, Jing Jing, Yuesong Pan, Ka Sing Wong, Yongjun Wang, Yilong Wang, on behalf of the Chinese IntraCranial AtheroSclerosis (CICAS) Study Group

**Affiliations:** 1 Department of Neurology, Beijing Tian Tan Hospital, Capital Medical University, Beijing, China; 2 Wayne State University School of Medicine, Detroit, Michigan, United States of America; 3 Department of Medicine and Therapeutics, Prince of Wales Hospital, Chinese University of Hong Kong, Hong Kong, China; St Michael's Hospital, University of Toronto, CANADA

## Abstract

**Background:**

The risk of a subsequent stroke following a minor stroke is high. However, there are no effective rating scales to predict recurrent stroke following a minor one. Therefore, we assessed the risk factors associated with recurrent ischemic stroke or transient ischemic attack (TIA) within one year of minor stroke onset in order to identify possible risk factors.

**Methods:**

Eight hundred and sixty-three non-cardioembolic ischemic stroke patients in the Chinese IntraCranial AtheroSclerosis Study that presented with minor stroke, defined as an admission National Institutes of Health stroke scale (NIHSS) score of ≤3, were consecutively enrolled in our study. Clinical information and imaging features upon admission, and any recurrent ischemic stroke or TIA within one year was recorded. Cox regression was used to identify risk factors associated with recurrent ischemic stroke or TIA within the year following stroke onset.

**Results:**

A total of 50 patients (6.1%) experienced recurrent ischemic stroke or TIA within one year of minor stroke onset. Multivariate Cox regression model identified lower admission NIHSS score (HR, 1.75; 95% CI, 1.32 to 2.33; *P*<0.0001), history of coronary heart disease (HR, 2.62; 95% CI, 1.17 to 5.86; *P* = 0.02), severe stenosis or occlusion of large cerebral artery (HR, 4.68; 95% CI, 1.87 to 11.7; *P* = 0.001), and multiple acute cerebral infarcts (HR, 2.61; 95% CI, 1.01 to 6.80; *P* = 0.05) as independent risk factors for recurrent ischemic stroke or TIA within one year.

**Conclusions:**

Some minor stroke patients are at higher risk for recurrent ischemic stroke or TIA. Urgent and intensified therapy may be reasonable in these patients.

## Introduction

The chance of a subsequent stroke following a minor stroke is high with a 90 day recurrence risk of 10% to 20% [1]. Of the patients with stroke deemed too mild (median NIHSS = 3) for thrombolytic therapy, 17% are either dependent or deceased upon hospital discharge [2]. In addition, 27% of mild or improving stroke patients who were not administered intravenous tissue plasminogen activator (tPA) were either deceased or refrained from discharged due to exacerbating neurological conditions [3]. Therefore, it is necessary to identify minor stroke patients with high recurrence risk. Meanwhile, there are many rating scales which predict recurrence of stroke in transient ischemic attack (TIA) patients such as ABCD2 and ABCD3 score [4,5]; however, it is not effective in predicting recurrent stroke in minor stroke patients, and nor is the Essen Stroke Risk Score or the Stroke Prognosis Instrument II [6]. Compared to TIA patients, minor stroke patients may have different risk factors associated with recurrent ischemic stroke or TIA. Besides, most previous studies [5,7,8] defined TIA according to the classic definition, and therefore some minor stroke patients may be diagnosed as TIA according to the classic definition of TIA. In light of this, our study only enrolls minor stroke patients and aims to elucidate the factors associated with recurrent ischemic stroke or TIA within one year of minor stroke onset.

## Methods and Materials

### Ethics Statement

This study was approved by the ethics committee of the Beijing Tian Tan Hospital of Capital Medical University and was performed in accordance with the guidelines of the Helsinki Declaration. After ethical approval of Tian Tan Hospital was obtained and approved by the other 21 participating hospitals, the ethical approval took effect automatically in each center. All patients or their legal representatives provide their written informed consent form.

### Subjects

Eight hundred and sixty-three non-cardioembolic ischemic stroke patients from the Chinese IntraCranial AtheroSclerosis Study (ICAS) who presented with a minor stroke were enrolled the study. ICAS is a prospective, multicenter, cohort, hospital based study, a total of 2864 noncardioembolic ischemic stroke or TIA patients in 22 general hospitals covering a wide geographic area in China were enrolled consecutively. Upon admission, baseline data, including age, gender, medical history, physical examination, laboratory tests, and electrocardiography were collected. All patients underwent detailed radiographic evaluation, including cranial magnetic resonance (MR) scan, 3 dimensional time of flight MR angiography (3D TOF MRA), and duplex color Doppler ultrasound or contrast enhanced MRA (CEMRA). Full details of the design of the ICAS were published previously [9].

**Fig 1:**
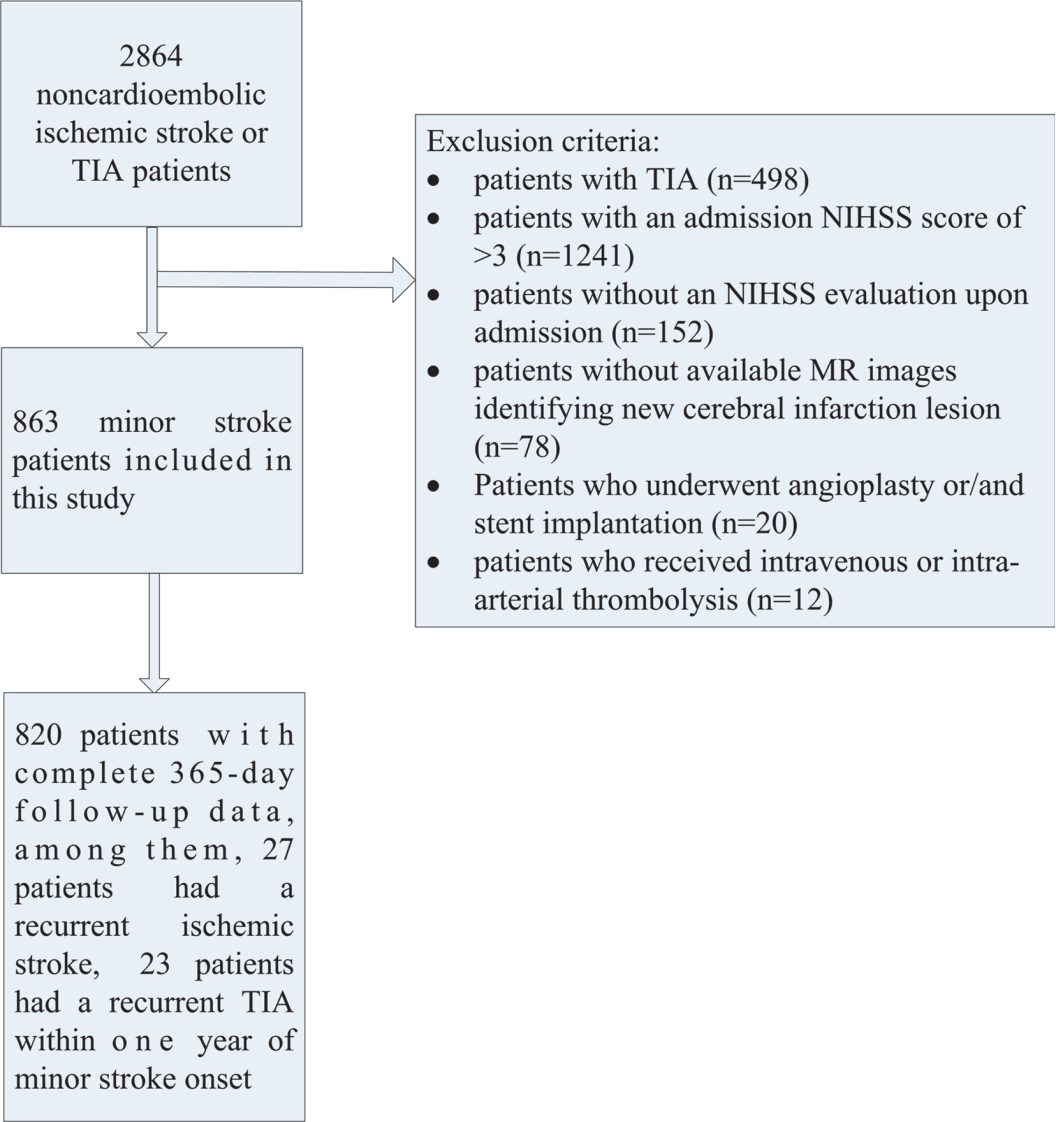
Flow chart of enrollment of study population. TIA, transient ischemic attack; NIHSS, National Institutes of Health stroke scale.

Inclusion criteria of ICAS included onset of ischemic stroke or TIA within 7 days and age of 18 to 80 years. Excluded patients included the clinically unstable, those that required close monitoring, the moribund, ones that were disabled before admission (modified Rankin scale >2), or those who were physically or subjectively unable to comply with MR examination. All patients underwent Electrocardiography and Echocardiogram, patients with cardioembolic risk factors (including atrial fibrillation, valvular heart disease, postcardiac valve replacement, etc.) were excluded to rule out cardioembolic stroke patients. Additional exclusions in this subgroup of minor stroke patients included patients with TIA, patients with an admission NIHSS score of >3 or without an NIHSS evaluation upon admission, patients without available MR images identifying new cerebral infarction lesion, patients who underwent angioplasty or/and stent implantation, and patients that received intravenous or intra-arterial thrombolysis. In essence, 863 of the 2864 patients that presented with a minor stroke were included in our study (Fig. 1).

The clinical and imaging information of these patients such as age, gender, past history, condition of antithrombotics treatment, etiologic subtype of ischemic stroke, topographical distribution of infarct lesions, and stenotic degree of responsible cerebral artery were evaluated and the associated risk factors for recurrent ischemic stroke or TIA within one year were analyzed.

Minor stroke patients were defined as having an admission NIHSS score of ≤3 and diffusion weighted imaging (DWI) hyperintensity consistent with acute cerebral infarct. Trained research personnel at Beijing Tian Tan Hospital contacted patients at 3, 6, and 12 months after stroke onset via telephone. The primary outcome was a recurrent ischemic stroke or TIA. All recurrent ischemic stroke or TIA associated with re-hospitalization was verified at the index hospitals based on the medical records combined with CT or MR images. An experienced stroke neurologist reviewed the patients’ medical document and all pertinent brain images to ensure a reliable diagnosis. In case of a suspected recurrent cerebrovascular event without hospitalization, the decision was made conjointly by the research coordinator (stroke neurologist) and principle investigator (Yongjun Wang). Each case fatality and cause of death was either confirmed on the death certificate from local citizen registry or from the attended hospital.

Recurrent ischemic stroke was defined as a sudden focal neurological deficit sustained for a duration of >24 hours suggesting a new ischemic event which was verified by cranial CT or MRI [10]. A recurrent TIA was defined as a new focal neurological deficit sustained for a duration of <24 hours caused by ischemia in the brain or retina.

### Brain MRI

All 863 patients underwent cranial MRI within 4 days of stroke onset, including 3D TOF MRA on a 3.0 or 1.5 T MR scanner with 1mm slices thickness, and axial T2 weighted, T1 weighted imaging, fluid attenuated inversion recovery sequences, and DWI performed with 5 mm slices thickness. Images were viewed using software (RadiAnt DICOM Viewer1.0.4.4439, Medixant Ltd, poznan, Poland).

The stenotic degree of intracranial vessels, and extracranial carotid and vertebral vessels were examined using 3D TOF MRA, and duplex color Doppler ultrasound or CEMRA, respectively. Responsible artery for acute cerebral infarct was defined as intracranial or extracranial artery responsible for acute cerebral infarct according to the distribution characteristic of infarct lesions and examination results of MRA and CEMRA or color Doppler ultrasound. The degree of responsible intracranial stenosis on MRA was calculated using the published method for the Warfarin–Aspirin Symptomatic Intracranial Disease Study [11]. The degree of responsible extracranial artery stenosis was estimated with ultrasonographic examination according to the published diagnostic criteria or calculated according to North American Symptomatic Carotid Endarterectomy Trial (NASCET) criteria by CEMRA [12,13]. All measurements were made using Wiha DigiMax Digital Calipers 6’ (Germany) with a resolution of 0.01 to 0.03 mm for 0 to 100 mm. We assessed the following responsible arterial segments: bilateral internal carotid artery (ICA), anterior cerebral artery (ACA) A1/A2, middle cerebral artery (MCA) M1/M2, posterior cerebral artery (PCA) P1/P2, vertebral artery, and basilar artery (BA). According to the severity of the stenosis, we classified the responsible cerebral vessels into 4 groups: <50% or no stenosis, 50% to 69% stenosis, 70% to 99% stenosis, and occlusion groups. Severe stenosis or occlusion of the responsible large cerebral artery (LCA) was defined as either an occlusion or a stenosis of ≥70% of the responsible ICA, proximal (M1 or M2) MCA, or basilar artery (BA).

**Fig 2:**
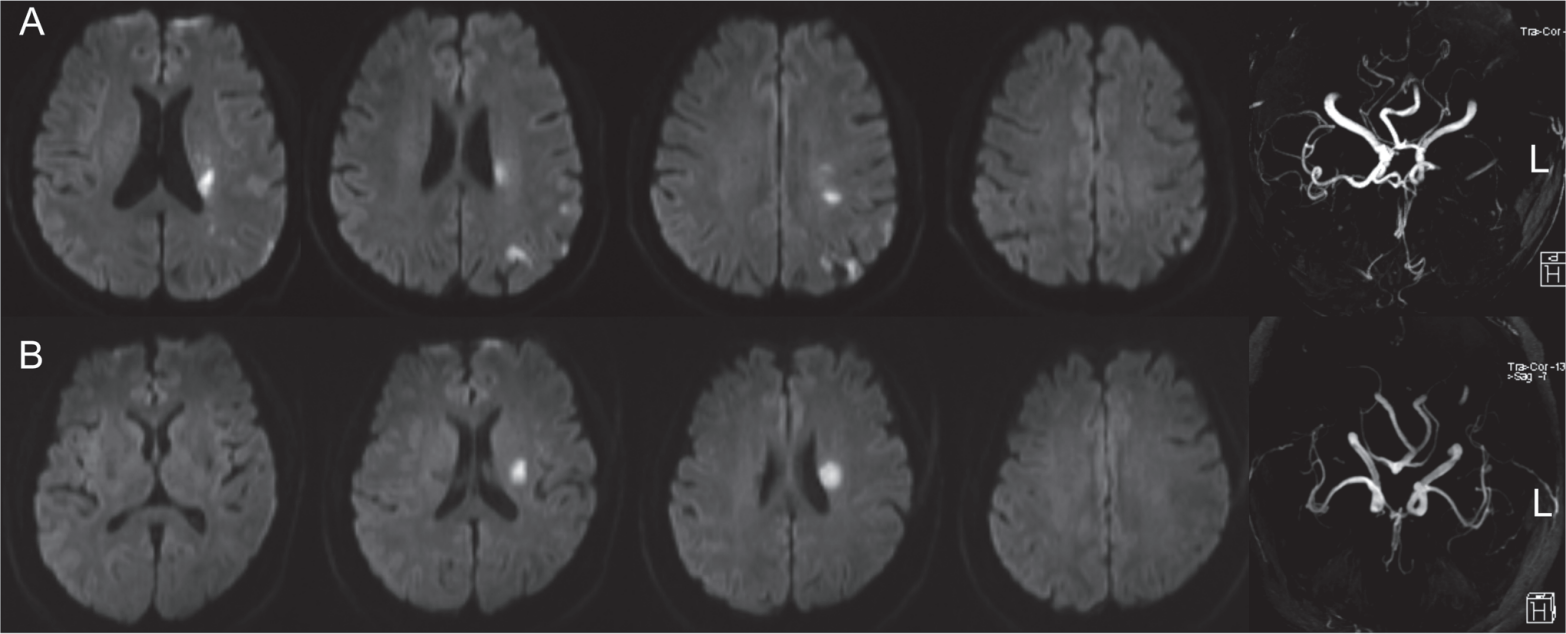
The topographical distribution of infarct lesions and etiologic subtype of minor stroke. A. minor stroke with occlusion of left middle cerebral artery (MCA) and multiple cerebral infarcts which located in cortex, internal watershed, and posterior cortical watershed, the etiologic subtype was considered as large artery atherosclerosis. B. minor stroke with single subcortical infarction in the left MCA perforator territory but without parent artery disease, the etiologic subtype was considered as small artery occlusion.

Etiologic subtypes of ischemic stroke were classified according to the Stop Stroke Study Trial of Org 10172 in Acute Stroke Treatment (SSS-TOAST) classification criteria [14]. Topographical distribution of acute infarct lesions (including single or multiple acute infarcts, watershed infarcts, small cortical infarct, and territorial infarct) were evaluated (Fig. 2). Multiple acute cerebral infarcts was defined as ≥2 separate lesions that were hyperintense on DWI. Watershed infarcts were classified as internal watershed infarcts (IWS) and cortical watershed infarcts (CWS). IWS was defined as rosary-like pattern of infarcts arranged in a linear fashion parallel to the lateral ventricle and located in the centrum semiovale or corona radiata. CWS are distinguished as anterior cortical watershed infarcts (ACWS) and posterior cortical watershed infarcts (PCWS) using the method described in the previous study [15]. With the exception of watershed, small cortical infarct was defined as cortical infarct with a maximum diameter of <2 cm. Territorial infarct was defined as a large ischemic lesion with a maximum diameter of ≥2 cm involving the cerebral cortical and subcortical structure in one or more major cerebral artery territories [16].

Two radiologists blinded to the clinical details assessed the MRI scans. Consensus from disagreements were reached through discussion.

### Clinical Data Assessment

The clinical information included age, gender, hypertension (defined as a history of hypertension or diagnosis at discharge), diabetes mellitus (defined as a history of diabetes mellitus or diagnosis at discharge), hyperlipidemia (defined as low-density lipoprotein cholesterol of ≥2.6 mmol/L upon admission, a history of hyperlipidemia, having undergone lipid-lowering treatments, or diagnosed at discharge), history of stroke (including ischemic and hemorrhagic stroke), history of coronary heart disease (defined as a history of myocardial infarction or angina pectoris), and an NIHSS score upon admission. Additionally, current or previous smokers (patients who continuously smoked ≥1 cigarette a day for 6 months), and heavy drinkers (patients who consumed >2 drinks/day on average for men or >1 drink/day on average for women, and a standard drink was defined as a glass of wine, a bottle of beer, or a shot of spirits, approximating 10 to 12 g of ethanol [17]) were noted. Condition of antithrombotics treatment within 48 hours of admission, at discharge, and within 1 year after stroke onset were also recorded.

### Statistical Analyses

Continuous variables with non-normal distribution were summarized as median (interquartile range). Categorical variables were presented as numbers (percentages). Mann-Whitney U test and Chi-squared (χ^2^) test were used to identify baseline differences in clinical and imaging variables between patients with or without recurrent stroke or TIA. The risk of subsequent stroke or TIA in relation to each variable was determined by multivariable Cox regression analysis. The regression model included the time from stroke onset to a recurrent stroke or TIA as response, and clinical and imaging predictors of recurrence with a univariate *P* value <0.05 as independent variables. All probability values were 2-tailed, with a *P*<0.05 considered as statistically significant. All analyses were performed using SAS Version 9.1 (SAS Institute, Inc, Cary, NC).

## Results

We excluded 498 patients with TIA, 1241 patients with an admission NIHSS score of >3, 152 patients without an NIHSS evaluation upon admission, 78 patients without available MR images identifying new cerebral infarction lesion, 14 patients who underwent stent implantation, 1 patient who underwent angioplasty, 5 patient who underwent angioplasty and stent implantation, and 12 patients who received intravenous or intra-arterial thrombolysis. In essence, a total of 863 minor stroke patients (609 men, 254 women) with a mean age of 61.1±11.2 years (range, 29 to 80 years) were recruited (Table 1). Among the 863 patients, minor stroke in 501 patients (58.1%) were due to large artery atherosclerosis (LAA), while 362 (41.9%) were due to small artery occlusion (SAO) according to SSS-TOAST classification. Complete 365-day follow-up was available in 820 patients. The remaining 13 patients were deceased (2 of which passed away from recurrent ischemic stroke), while the other 30 patients were lost to follow up during the 365-day period.

**Table 1:** Univariate Analysis for Prediction Factors of Recurrent Ischemic Events in one year after minor stroke.

Variables	Total (n = 863)	No recurrence(n = 813)	Recurrence(n = 50)	P value
**Demographics**				
Age, median (IQR), y	61 [53,71]	61 [53,71]	62 [54,70]	0.83
Age ≥65 years	354(41.0)	336(41.3)	18(36.0)	0.46
Male	609(70.6)	572(70.4)	37 (74.0)	0.58
**Vascular Risk factors**				
Smoking	436(50.5)	409(50.3)	27 (54.0)	0.61
Drinking	313(36.3)	292 (35.9)	21 (42.0)	0.39
Hypertension	659(76.4)	622(76.5)	37(74.0)	0.69
Diabetes mellitus	301(35.0)	289(35.7)	12(24.0)	0.10
Hyperlipidemia	655(75.9)	617(75.9)	38 (76.0)	1.00
CHD	64(7.4)	56(6.9)	8 (16.0)	0.02
History of stroke	204(23.6)	192(23.6)	12(24.0)	0.95
History of ischemic stroke	195(22.6)	183(22.5)	12 (24.0)	0.81
**Clinical and Imaging Features**				
Admission NIHSS,median (IQR)	2[1,3]	2[1,3]	1[0,2]	<0.0001
Day from onset to admission	2[1,3]	2[1,3]	1[1,3]	0.39
LAA subtype*	501(58.1)	462 (56.8)	39 (78.0)	0.003
Responsible artery stenosis ≥70%	388(45.0)	350 (43.1)	38 (76.0)	<0.0001
Severe stenosis or occlusion of LCA	301(34.9)	267 (32.8)	34 (68.0)	<0.0001
Multiple acute cerebral infarcts	320(37.1)	287(35.3)	33 (66.0)	<0.0001
Watershed infarcts	238(27.6)	215 (26.4)	23 (46.0)	0.003
IWS	177(20.5)	161 (19.8)	16 (32.0)	0.04
ACWS	107(12.4)	95 (11.7)	12 (24.0)	0.01
PCWS	141(16.3)	130(16.0)	11 (22.0)	0.27
Territorial infarct	121(14.0)	113 (13.9)	8 (16.0)	0.68
Small cortical infarct	247(28.6)	227 (27.9)	20 (40.0)	0.07
**Performance measures**				
Early Antithrombotics after admission	836(97.8)	787 (97.6)	49 (100.0)	0.28
Antithrombotics at discharge	816(95.6)	771 (95.9)	45 (90.0)	0.05
Aspirin only	546(63.3)	522 (64.2)	24 (48.0)	0.02
Clopidogrel only	235(27.2)	216 (26.6)	19 (38.0)	0.08
Cilostazol only	7(0.8)	7 (0.9)	0 (0)	1.00
Ticlopidine only	2(0.2)	2 (0.2)	0 (0)	1.00
Warfarin only	2(0.2)	2 (0.2)	0 (0)	1.00
Aspirin plus clopidogrel	24(2.8)	22 (2.7)	2 (4.0)	0.65
Antithrombotics in 1 year	573(66.4)	544 (66.9)	29 (58.0)	0.20
Aspirin only	466(54.0)	444 (54.6)	22 (44.0)	0.14
Clopidogrel only	85(9.8)	80 (9.8)	5 (10.0)	0.97
Ticlopidine only	1(0.1)	1 (0.1)	0 (0)	1.00
Aspirin plus clopidogrel	19(2.2)	17 (2.1)	2 (4.0)	0.30
Aspirin plus dipyridamole	1(0.1)	1 (0.1)	0 (0)	1.00
Clopidogrel plus warfarin	1(0.1)	1 (0.1)	0 (0)	1.00

IQR, interquartile range; CHD, coronary heart disease; NIHSS, National Institutes of Health stroke scale; LAA, large artery atherosclerosis; LCA, large cerebral artery; IWS, internal watershed infarcts; ACWS, anterior cortical watershed infarcts; PCWS, posterior cortical watershed infarcts; Data are n (%) unless otherwise indicate.

*It is contrary to small artery occlusion subtype of ischemic stroke according to Stop Stroke Study Trial of Org 10172 in Acute Stroke Treatment (SSS-TOAST) classification criteria.

Overall fifty (6.1%) patients (including the 2 patients that passed away from recurrent ischemic stroke) developed recurrent ischemic stroke or TIA within a year of minor stroke onset. Of them, 27 patients had a recurrent ischemic stroke, and 23 patients had a recurrent TIA. Meanwhile, 16 patients developed recurrent ischemic stroke or TIA within the first 2 days after stroke onset, 29 patients developed recurrent ischemic stroke or TIA within the first 7 days, and 34 patients developed recurrent ischemic stroke or TIA within the first 14 days.

In univariate analysis (Table 1), patients with recurrence of ischemic stroke or TIA had a higher rate of CHD (8 [16.0%] vs 56 [6.9%], p = 0.02), LAA subtype stroke (39 [78.0%] vs 462 [56.8%], p = 0.007), responsible artery stenosis ≥70% (38 [76.0%] vs 350 [43.1%], p<0.0001), severe stenosis or occlusion of responsible LCA (34 [68.0%] vs 267 [32.8%], p<0.0001) than those without recurrence. Patients with recurrence had a lower median NIHSS at admission (1 vs 2, p<0.0001) than those without recurrence. Finally, those with recurrence experienced multiple acute cerebral infarcts (33 [66.0%] vs 287 [35.3%], p<0.0001), watershed infarcts (23 [46.0%] vs 215 [26.4%], p = 0.003), IWS (16 [32.0%] vs 161 [19.8%], p = 0.04), and ACWS (12 [24.0%] vs 95 [11.7%], p = 0.01) more frequently than those without any recurrence. There was no differences between patients with and without recurrence in age, gender, smoking, drinking, hypertension, diabetes, hyperlipidemia, history of stroke, and median time from onset to admission.

When adjusted for age, gender, vascular risk factors, and antithrombotics treatment at discharge, multivariate Cox regression model identified a lower admission NIHSS score (HR, 1.75; 95% CI, 1.32 to 2.33; *P*<0.0001), history of CHD (HR, 2.62; 95% CI, 1.17 to 5.86; *P* = 0.02), severe stenosis or occlusion of LCA (HR, 4.68; 95% CI, 1.87 to 11.7; *P* = 0.001), and multiple acute cerebral infarcts (HR, 2.61; 95% CI, 1.01 to 6.80; *P* = 0.05) as independent predictors of recurrent ischemic stroke or TIA within one year (Table 2).

**Table 2:** Multivariable COX Regression Analysis for Prediction Factors of Recurrent Ischemic Events in 1 Year after Minor Stroke.

Variables	HR* (95% CI)	P value
Age ≥65 years	0.83(0.44–1.58)	0.58
Male	0.94(0.42–2.10)	0.88
Smoking	0.76(0.34–1.71)	0.51
Drinking	1.41(0.66–3.05)	0.38
Hypertension	0.90(0.46–1.75)	0.76
Diabetes mellitus	0.57(0.29–1.14)	0.11
Hyperlipidemia	1.16(0.57–2.33)	0.69
CHD	2.62(1.17–5.86)	0.02
Admission NIHSS	0.57(0.43–0.76)	<0.0001
LAA subtype^†^	0.44(0.14–1.39)	0.16
Severe stenosis or occlusion of LCA	4.68(1.87–11.7)	0.001
Multiple acute cerebral infarcts	2.61(1.01–6.80)	0.05
Watershed infarcts	1.46(0.50–4.27)	0.49
Internal watershed infarcts	0.40(0.16–1.05)	0.06
ACWS	1.19(0.51–2.77)	0.69
No antithrombotics at discharge	1.57(0.58–4.21)	0.37

CHD, coronary heart disease; NIHSS, National Institutes of Health stroke scale; LAA, large artery atherosclerosis; LCA, large cerebral artery; ACWS, anterior cortical watershed infarcts; HR, hazards ratio; CI, confidence interval.

*Multivariable COX regression analysis adjusted for age, gender, stroke risk factors, admission NIHSS score, SSS-TOAST subtypes, stenosis or occlusion of LCA, topographical distribution of acute infarct lesions, and antithrombotics treatment at discharge.

^†^It is contrary to small artery occlusion subtype of ischemic stroke.

## Discussion

In our study, recurrence rate of ischemic stroke and TIA within one year was 3.3% and 2.8%, respectively. These values are lower than those previously reported [1,10]. A systematic review found that differences between studies with regards to early risks of stroke after TIA could largely be accounted for by differences in study method, setting, and treatment [18]. Thus, we infer that differences in study method, setting, and treatment may also result in the differences observed in risks of recurrent stroke or TIA after minor stroke. Firstly, almost all studies found that majority of recurrent stroke or TIA occurred within 2 days after onset [18]. However, the median time from onset to admission was 2 days in our study, which is longer than the median arrival time of 12 hours in Ois’s study [10]. This may in part explain the lower recurrence rate in our study. Secondly, all patients enrolled in our study were hospitalized, 816 patients (95.6%) received antithrombotic therapy, and 24 paitents (2.8%) received dual antithrombotic therapy of aspirin and clopidogrel. Combined administration of aspirin and clopidogrel was found to be superior to aspirin alone in reducing the risk of recurrent stroke in minor stroke patients [19], therefore this therapy also may have contributed to the lower recurrence rate. Besides, most previous studies [1,10,19,20] about recurrence of minor stroke included cardioembolic stroke, this may explain the higher recurrence rate in previous studies in some degree.

The goal of our study was to verify the risk factors for recurrence of ischemic stroke or TIA in minor stroke patients. The results indicate that a lower admission NIHSS score, history of CHD, multiple acute cerebral infarcts, and severe stenosis or occlusion of LCA are significantly related to recurrence of ischemic stroke or TIA within one year when adjusted for other factors in multivariate Cox regression analysis. A previous study had found that ABCD2 score, Essen Stroke Risk Score and Stroke Prognosis Instrument II were not effective predictors of recurrent stroke following a minor stroke [6]. Therefore, minor stroke may have distinctive risk factors of recurrence. Additionally, findings of Otis et al. indicated that age, gender, hypertension, diabetes, and prior stroke were not associated with recurrence of ischemic stroke [10], which are consistent with our findings that the aforementioned variables are not associated with recurrent ischemic stroke or TIA.

Our study also found that the recurrence rate of ischemic stroke or TIA was 6.1% within one year of minor stroke (863 patients) onset, which is higher than the 3.9% recurrence rate of ischemic stroke or TIA in our study of stroke patients with an admission NIHSS score of ≥4 (1241 patients). We also found that minor stroke patients with lower admission NIHSS score had a higher recurrence rate of ischemic stroke or TIA, an association which remained significant despite adjustment of other factors. Therefore, a lower admission NIHSS score is an independent risk factor of recurrent stroke or TIA in minor stroke patients. Perhaps the reason for this is that minor stroke patients with a lower NIHSS score have not reached the highest severity of stroke upon admission thus permitting the plaque to remain highly thrombogenic [21], which may result in a higher risk of recurrence. Additionally, minor stroke patients in our study with clinical symptoms or signs lasting <24 hours and zero NIHSS score at admission may be diagnosed as TIA in some other studies. TIA patients were found to have a higher risk of recurrent stroke than minor stroke patients in previous studies [22], which perhaps explains the higher recurrence rate in patients with a lower NIHSS score. In other words, minor stroke patients, especially patients with zero NIHSS score, should not be neglected, but rather undergo proactive treatment and management.

Previous studies had found that CHD or asymptomatic coronary stenosis is the strong predictor of stroke [23,24]. Stroke of carotid origin and carotid atherosclerosis are CHD risk equivalents [25], so coronary artery disease and cerebrovascular diseases are highly related. Our study found that history of CHD was significantly associated with recurrence of ischemic stroke or TIA after adjustment of other risk factors, therefore, CHD can be regarded as an independent risk factor.

In our study, minor stroke patients with occlusion or ≥70% stenosis of ICA, M1 or M2 of MCA, or BA had a higher recurrence rate of cerebral ischemic events, which is fairly consistent with finding of a previous study indicating that severe symptomatic extra or intracranial arterial disease was independently associated with 7-day and 90-day stroke recurrence in minor stroke patients [10]. In fact, severe stenosis or occlusion of a symptomatic intracranial or extracranial artery may cause misery perfusion, exhausted vasodilatory capacity, or microemboli [26–28], thus making severe stenosis or occlusion an intuitive independent risk factor for subsequent stroke or TIA.

We also found that watershed infarcts, IWS, and ACWS were significantly associated with recurrent ischemic stroke or TIA in univariate Cox regression analysis. However, when adjusted for severe stenosis or occlusion of LCA, the relationship was not significant. This is perhaps because patients with watershed infarcts, IWS, or ACWS frequently exhibited ≥70% stenosis of ICA or MCA.

Our study also found that minor stroke patients with multiple acute cerebral infarcts had a higher risk of recurrent ischemic stroke or TIA. Consistently, Ay et al. found that multiple acute infarcts was an independent predictor of 90-day risk of stroke [25]. Multiple new cerebral infarcts are often caused by small emboli from an unstable source such as rupture of plaque in a large artery atherosclerosis [29], while isolated subcortical or deep lesions are often caused by local small artery disease or parent artery plaque occluding penetrating artery [30]. Perhaps this may explain why minor stroke patients with multiple new cerebral infarcts exhibit a higher recurrence risk than patients with a single new cerebral infarct. It's worth noting that multiple acute cerebral infarcts were nonetheless still deemed an independent risk factor of recurrence despite adjustment of the responsible cerebral artery stenosis ≥70%. Additionally, a previous study has found that the majority of unstable plaque were in arteries of <70% stenosis [31], thus providing a possible explanation as to why multiple new cerebral infarcts are an independent risk factor of stroke or TIA recurrence.

We found that 58% of the recurrences (29 patients) of ischemic stroke or TIA occurred within the first 7 days after stroke onset, thus necessitating urgent and intensive therapy. Wong et al. found that combination therapy with clopidogrel and aspirin for 7 days is more effective than aspirin alone in reducing microembolic signals in patients with symptomatic ICA or MCA stenosis [32]. In a recent randomized placebo-controlled trial, the combined administration of clopidogrel and aspirin for 21 days after TIA or minor stroke onset is superior to aspirin alone for reducing the risk of stroke in the first 90 days without increasing the risk of hemorrhage [19], hence providing a promising therapeutic avenue to reduce early recurrence rate in minor stroke patients. Additionally, pooled analysis has shown that endarterectomy can reduce the risk of stroke in patients with 70% or greater symptomatic ICA stenosis [33]. Therefore, minor stroke patients with mulitiple new infarcts or ≥70% symptomatic ICA stenosis may benefit from combination therapy with clopidogrel and aspirin or endarterectomy, which may reduce the risk of recurrent ischemic stroke or TIA.

### Strengths and Limitations

The strengths of our study include the large sample size of minor stroke patients, and our use of standardized methods to evaluate the patients’ multiple imaging features such as SSS-TOAST stroke etiologic subtype, degree of responsible artery stenosis, and distribution patterns of cerebral infarctions. A limitation of our study is that some extracranial artery stenosis were estimated with ultrasonographic examination, while some others were calculated according to NASCET criteria by CEMRA, which may be the source of some differences when compared.

## Conclusions

In summary, our data show that patients with a lower admission NIHSS score, history of CHD, multiple acute cerebral infarcts, and severe stenosis or occlusion of LCA are at higher risk for recurrent ischemic stroke or TIA within one year of minor stroke onset; therefore, urgent and intensified therapy may be reasonable for prevention of recurrent ischemic stroke or TIA in such patients.

## Supporting Information

S1 Dataset This is the CICAS database of 863 minor stroke patients. (RAR)
